# The impact of self-stigma on functioning among remitted patients with bipolar disorder

**DOI:** 10.1192/j.eurpsy.2022.1022

**Published:** 2022-09-01

**Authors:** R. Jenhani, S. Ellouze, D. Bougacha, F. Znaidi, R. Ghachem

**Affiliations:** 1 Razi hospital, Psychiatry B, Manouba, Tunisia; 2 Hedi Chaker University Hospital, Psychiatry B, Sfax, Tunisia; 3 Razi hospital, B, Manouba, Tunisia; 4 Razi Hospital, Psychiatry B, Manouba, Tunisia

**Keywords:** functioning, bipolar disorder, self-stigma

## Abstract

**Introduction:**

Self-stigma is widespread in patients with bipolar disorder, with many consequences for family, social and occupational functioning, as well as treatment adherence.

**Objectives:**

The aim of this study was to evaluate self-stigma in remitted patients with bipolar disorder and to investigate its impact upon functioning.

**Methods:**

We conducted a cross-sectional, descriptive, and analytical study of 61 patients with bipolar disorder. Euthymia was verified using the Hamilton scale for depression and the Young scale for mania. We used the Internalized Stigma of Mental Illness (ISMI) to evaluate self-stigma, the Functioning Assessment Short Test (FAST) to assess functioning.

**Results:**

The mean age of patients was 43.4 years. The sex ratio was 2.4. The mean score on the ISMI was 2.36. More than half of our patients (59%) were self-stigmatized. Regarding functioning, a global impairment was noted in more than two thirds of the patients (71%). Occupational functioning was the most affected area (82%). Patients with higher self-stigma scores had significantly more impaired functioning (p<10^-3^). To decompose the relationship between stigma and functioning into more specific spheres, we found that all scores on the different domains of functioning were associated with a significantly higher mean self-stigma score.

**Conclusions:**

The relationship between self-stigma and functioning seems to be bidirectional. Therefore, improved social functioning could reduce self-stigma and improve self-esteem.

**Disclosure:**

No significant relationships.

